# Supplementary material for: From Deer-to-Deer: SARS-CoV-2 is efficiently transmitted and presents broad tissue tropism and replication sites in white-tailed deer
Source: PLoS Pathog. 2022 Mar 21;18(3):e1010197. doi: 10.1371/journal.ppat.1010197 (PMC8970504; doi:10.1371/journal.ppat.1010197)
Supplement: S1 Table — (DOC) [file ppat.1010197.s001.doc]

| Position | Amino Acid Change | CDS Codon Number | Change | product | Protein Effect |
| --- | --- | --- | --- | --- | --- |
| 203 |  |  | C -> T |  |  |
| 3264 | T -> I | 1000 | C -> T | orf1ab polyprotein | Substitution |
| 4100 |  | 1279 | T -> C |  | None |
| 4173 | T -> I | 1303 | C -> T |  | Substitution |
| 7303 |  | 2346 | C -> T |  | None |
| 9430 |  | 3055 | C -> T |  | None |
| 11050 |  | 3595 | C -> T |  | None |
| 12073 |  | 3936 | C -> T |  | None |
| 12076 |  | 3937 | C -> T |  | None |
| 18740 | D -> G | 6159 | A -> G |  | Substitution |
| 23929 |  | 789 | C -> T | surface glycoprotein | None |
| 26469 |  | 75 | C -> T | envelope protein | None |
| 26546 |  | 8 | T -> C | membrane glycoprotein | None |
| 27509 | T -> I | 39 | C -> T | ORF7a protein | Substitution |
| 27577 |  | 62 | C -> T |  | Truncation |
| 28253 |  | 120 | C -> T | ORF8 protein | None |
| 28603 |  | 110 | C -> T | nucleocapsid phosphoprotein | None |
| 29679 |  |  | C -> T |  |  |

Supplementary Table 1. Low frequency mutations observed during SARS-CoV-2 replication in WTD.
